# The Pattern of Heparin Dosing as Venous Thromboembolism Prophylaxis in Adult Underweight Patients Admitted to Critical Care Units at a Tertiary Hospital

**DOI:** 10.7759/cureus.31717

**Published:** 2022-11-21

**Authors:** Nouf Al Harthy, Felwah Al Harthi, Shmeylan Al Harb, Najwa Al Dughayem, Khoulod Al Harthi, Jawaher Gramish

**Affiliations:** 1 Pharmaceutical Care, King Abdulaziz Medical City, Riyadh, SAU; 2 Emergency Medicine, King Abdulaziz Medical City, Riyadh, SAU; 3 Pharmacy, College of Pharmacy, King Saud Bin Abdulaziz University for Health Sciences, Riyadh, SAU; 4 Pharmacy, Pharmaceutical Care, King Abdulaziz Medical City, Riyadh, SAU

**Keywords:** tertiary medical hospital in riyadh, critical care units, adult patients, venous thromboembolism prophylaxis, heparin administration

## Abstract

Background: Venous thromboembolism (VTE) is one of the causes of hospital-related deaths in critically ill patients. Guidelines recommended VTE prophylaxis with standardized, fixed doses for most patients. The underweight population has limited data to guide the appropriate drug and dosing regimen.

Objective: The aim of this study was to describe the pattern of VTE prophylaxis dose regimens for underweighted critically ill adult patients and the prevalence of associated VTE and bleeding.

Methods: This study is a retrospective cohort study, conducted at the King Abdulaziz Medical City, Riyadh, Saudi Arabia. It included all critical patients admitted to the intensive care units (ICUs) and were above 14 years old with weight less than 50 kg or body mass index (BMI) of 18.5 kg/m^2^ or less, and were on heparin as VTE prophylaxis for more than 72 h from January 2016 until January 2020.

Results: After screening 270 patients, only 40 patients were included in this study according to our inclusion and exclusion criteria. Only six patients (15%) received VTE prophylaxis as an adjusted dose of heparin 2500 U Q12, while the rest of the patients were taking standard dosing of heparin; 5000 U Q12 was given to 21 (52.50%) patients, and 5000 U Q8 was given to 13 (32.50%) patients. None of the adjusted doses developed any complications such as VTE or bleeding. There was no significant difference compared with the standard dose group.

Conclusions: In this study, we described the pattern of heparin doses as VTE prophylaxis in underweight patients. We also compared the standard dosing and adjusted dosage of VTE prophylaxis on underweight patients and any complications. There was no significant difference in the complications outcome or benefits between the two groups.

## Introduction

Venous thromboembolism (VTE) is a complex disease in which clinical management is still challenging [1]. Notably, recent evidence found that more than half of hospitalized patients are at risk of thromboembolism. VTE is one of the most common causes of hospital-related deaths and the most preventable. The rate of VTE is up to 80% in patients who are not on any prophylaxis methods. Studies have proven the rate of VTE is higher in hospitalized critically ill patients and can occur in approximately 44% of critically ill patients. Especially pulmonary embolism (PE), which has been reported to be around 12% of patients despite the form of VTE prophylaxis [2-3].

Current guidelines from the American College of Chest Physicians, based on evidence from clinical trials, recommended VTE prophylaxis with standardized fixed doses for either subcutaneous heparin, low-molecular-weight heparin, or other anticoagulant medication for all critically ill patients without any contraindication or special considerations for clinical factors [4-5]. These standard doses are used in the majority of patients; however, some patients who have co-morbidity, liver and kidney dysfunction, extreme weight differences, and other current conditions have the potential for either overdosing or underdosing due to altered pharmacokinetics and dynamics which will lead us to face more complications like thrombosis or bleeding [1, 6].

In specific, extreme weight has limited data to guide us to the appropriate drug and dosing regimen, especially in the underweight population. Underweight patients were also primarily underrepresented in the existing prospective clinical trials of VTE prophylaxis in critically ill patients. Due to low body weight, there may be differences in the volume of distribution and renal clearance because of low muscle mass and the lack of adipose tissue; these potential factors play a significant role in pharmacokinetics that produces different results in variability, safety, and efficacy for these populations. Primarily, low body weight patients were associated with increased risks of bleeding despite receiving anticoagulants. As a result, no available pharmacological prophylaxis regimens for critically ill, underweight patients were established until today [6-8].

## Materials and methods

Study design and technique

The study design was a single-center, retrospective cohort study conducted on eligible patients who met the inclusion criteria and were admitted to the intensive care units (ICUs) during the period from January 2016 until January 2020 at the King Abdulaziz Medical City, Riyadh, Saudi Arabia. Convenient sampling was used on all included patients, to report the percentage of underweight critically ill patients who received an adjusted dose of VTE prophylaxis vs. patients that received a standard dose of VTE prophylaxis. As well as, reporting the prevalence of clinically relevant new-onset thrombosis and bleeding events. It is noteworthy here that the causative disease that led to ICU admission in the study setting was mainly severe community-acquired pneumonia, sepsis, and congestive heart failure. However, other diseases were present among the recruited patients. 

Study setting and subjects

The study took place at the King Abdulaziz Medical City, Riyadh, Saudi Arabia. The study was approved by the Institutional Review Board at King Abdullah International Medical Research Center (KAIMRC) (approval number: RYD - 20 - 419812-15389/ Protocol number RC20/033/R). The study included all ICUs such as (ICU2, TICU, NCCU, IMCU, PRU, SICU, BICU, ICU10, MCICU, ACICU, and CCU). All patients admitted in one of the previous units, who were older than 14 years old with a body mass index (BMI) of 18.5 kg/m2 or less as well as a weight of 50 kg or less, and were on VTE prophylaxis for more than 72 h were included. However, patients who lasted less than 72 h in any ICU divisions were excluded from this study.

Data collection methods, instruments used, and measurements

The investigators received an e-mail from the Clinical Information Manager (CIM), which included all patients admitted to ICUs with specific information [patient name, medical recorded number (MRN), most responsible physician (MRP), and lastly under which specialty service]. Inclusion and exclusion criteria were applied to each patient. Conforming all the above information, we started to fill our data collection sheet with thrombosis or bleeding events in the first month after starting VTE prophylaxis, as well as demographic and clinical variables that were collected from the electronic medical records via manual chart review using a standardized electronic data collection tool by a single reviewer.

Our definitions for some important variables collected in these patients were as follows:

- A VTE event was defined as any new deep vein thrombosis (DVT) identified by contrast venography, compression ultrasound, or impedance plethysmography or a pulmonary embolism (PE) confirmed by pulmonary angiography, lung scintigraphy, or helical CT scan [5].

- Standard VTE dose for prophylaxis was defined as a regimen containing unfractionated heparin UFH 10000-22500 U/d [5].

- Adjusted-dose prophylaxis was defined as any regimen containing UFH <10000 U/d [5].

- Major bleeding is defined as a hemoglobin drop of at least 2 g/dL in 24 h, a transfusion of at least one unit of packed red blood cells, bleeding into a critical site (e.g., intracranial, intraocular, retroperitoneal, intra-articular, pericardial, or intramuscular with compartment syndrome), or fatal bleeding [7].

- Minor bleeding was defined as overt bleeding not meeting the criteria for major bleeding (e.g., gastrointestinal bleeding, hematuria, hematemesis, or hematochezia) [7].

Data management and analyses

Data management and analysis were done using the Statistical Package for the Social Sciences (SPSS). Categorical variables were expressed as a percentage along with the new onset of thrombosis and bleeding events. However, continuous variables with normal distribution were expressed as mean and standard deviation, and non-normal distribution was expressed as median (interquartile). Lastly, the comparison analysis between both groups who received an adjusted dose of VTE prophylaxis and who received the standard dose of VTE prophylaxis was expressed by using a Chi-square test. All statistical tests were declared significant at a level of 0.05 or less.

Ethical considerations

Both hard and soft copy data were and are still secure within national guard hospital affairs, and both patient privacy and confidentiality were assured with no identifiers collected.

## Results

Demographic and clinical characteristics

During the period from January 2016 to January 2020, around 270 patients were screened. As shown in Table 1, only 40 patients were included in this study according to our inclusion and exclusion criteria, males were more than females, 21 were male patients (52.50%), and 19 female patients (47.50%) with a mean weight of 42.11 kg, a mean BMI of 16.76 and lastly a mean age of 51.73 years old. Out of 40 patients, 22 (55%) had normal renal function and 14 (35%) had a glomerular filtration rate under 90, while only four (10%) were under hemodialysis. The majority of patients included were free of chronic diseases, no coagulopathy patients, none had hepatitis and hepatocellular carcinoma, only two (5%) had cirrhosis and five (12.5%) had a thyroid disorder. Only two had chronic liver disease, and seven (17.50%) had abnormal liver function tests (Table 1).

VTE dosing and complications

The results presented in Table 1 showed that upon admission, only six (15%) patients received adjusted VTE prophylaxis doses, while the rest of the patients were taking standard dosing; 5000 U Q12 was given to 21 (52.50%) patients and 5000 U Q8 was given to 13 (32.50%) patients. As a complication only one (2.50%) patient had an incident of developing PE in addition, DVT was also seen in one (2.50%) patient. The mean time for developing VTE since admission to ICU was 9.2 days. On the other hand, four (10%) had a history of dropping in hemoglobin which was managed by blood transfusion, but none of the 40 patients had major bleeding due to prophylaxis (Table 1). 

Home medication use of antiplatelet

The results in Table 1 showed that aspirin was the most frequently used by only 11 (27.50%) patients and clopidogrel was used by only two (5%) of the 40 patients. On the other hand, none of them used either ticagrelor, dipyridamole, or prasugrel.

**Table 1:** Demographics and clinical characteristics (n=40). LTFs, liver function tests; VTE, venous thromboembolism; Hg, hemoglobin; PE, pulmonary embolism; Wt, weight; BMI, body mass index; DVT, deep vein thrombosis; GFR, glomerular filtration rate; HD, hemodialysis

Characteristic		Overall	
		Frequency	Percent (%)
Gender	Male	21	52.50
	Female	19	47.50
Renal function	Normal	22	55.00
	GFR 45-90	9	22.50
	GFR 30-43	1	2.50
	GFR 15-29	4	10.00
	HD	4	10.00
Hepatitis	No	40	100.00
Cirrhosis	Yes	2	5.00
	No	38	95.00
Normal LFTs	Yes	33	82.50
	No	7	17.50
Hepatic cell carcinoma	No	40	100.00
Thyroid disorder	Yes	5	12.50
	No	35	87.50
Adjusted VTE dose prophylaxis	Yes	6	15.00
	No	34	85.00
Dose	2500 Q12	6	15.00
	5000 Q12	21	52.50
	5000 Q8	13	32.50
DVT	Yes	1	2.50
	No	39	97.50
PE	Yes	1	2.50
	No	39	97.50
Bleeding after VTE prophylaxis	No	40	100.00
Drop in Hg	Yes	4	10.00
	No	36	90.00
Blood transfusion	Yes	4	10.00
	No	36	90.00
Aspirin	Yes	11	27.50
	No	29	72.50
Clopidogrel	Yes	2	5.00
	No	38	95.00
Ticagrelor	No	40	100.00
Dipyridamole	No	40	100.00
Prasugrel and dipyridamole	No	40	100.00
Age		51.73 ± 24.24	
Wt		42.11 ± 6.16	
BMI		16.76 ± 2.05	

Association of dosing with complications 

As shown in Table 2, only six patients received the adjusted dose of 2500 U Q12, among these six patients, two were receiving aspirin and one was receiving clopidogrel. None of these six patients who received the adjusted doses develop any complications of VTE or bleeding. On the other hand, for patients who used the standard dosing, 21 of them received the 5000 U Q12, five of those 21 were using aspirin and only one was using clopidogrel. Among these 21 patients, only one developed PE, and three developed a drop in hemoglobin that led to blood transfusion. However, no major bleeding episodes were seen in this category. The same dose with more frequency of 5000 U Q8 was given to 13 patients, four of them were receiving aspirin only, however, only one had a complication of DVT and only one had a drop in hemoglobin that led to transfusion and also no major bleeding episodes were seen (Table 2). In conclusion, the adjusted dosing for low-weight patients did not give significant results compared to the standard dosing with regard to complications.

**Table 2:** Association variables with dose. DVT, deep vein thrombosis; PE, pulmonary embolism; VTE, venous thromboembolism; Hg, hemoglobin; Wt, weight

Characteristic	2500 Q12	5000 Q12	5000 Q8	p value
Adjusted VTE dose prophylaxis				<0.0001>
Yes	6 100.00	0 0.00	0 0.00	
No	0 0.00	21 100.00	13 100.00	
DVT				0.4750
Yes	0 0.00	0 0.00	1 7.69	
No	6 100.00	21 100.00	12 92.31	
PE				1.0000
Yes	0 0.00	1 4.76	0 0.00	
No	6 100.00	20 95.24	13 100.00	
Bleeding after VTE prophylaxis				
No	6 100.00	21 100.00	13 100.00	
Drop in Hg				1.0000
Yes	0 0.00	3 14.29	1 7.69	
No	6 100.00	18 85.71	12 92.31	
Blood transfusion				1.0000
Yes	0 0.00	3 14.29	1 7.69	
No	6 100.00	18 85.71	12 92.31	
Aspirin				0.7986
Yes	2 33.33	5 23.81	4 30.77	
No	4 66.67	16 76.19	9 69.23	
Clopidogrel				0.3808
Yes	1 16.67	1 4.76	0 0.00	
No	5 83.33	20 95.24	13 100.00	
Ticagrelor				
No	6 100.00	21 100.00	13 100.00	
Dipyridamole				
No	6 100.00	21 100.00	13 100.00	
Prasugrel and dipyridamole				
No	6 100.00	21 100.00	13 100.00	
Wt	36.50 ± 3.94	42.47 ± 6.42	44.12 ± 5.28	0.0359

## Discussion

Anticoagulant medication is a highly complicated medication to be prescribed especially when it is used as a prophylaxis regimen. When it comes to critically ill patients, it is a standard practice to prescribe an anticoagulant dose of heparin 5000 U Q12 or 5000 U Q8, however, with the developing knowledge and constant practice with these types of medications, different studies are extending the anticoagulant medication guideline regarding the weight scope [7]. Two studies that were established in 2012 and 2014 have implemented new dosing taking into consideration the weight of their patients. They have been investigating a new regimen for overweight patients to attain 7500 units of three times daily of UFH, twice daily of enoxaparin 40 mg, and a weight-based calculation of enoxaparin 0.5 mg/kg daily. Some researchers proposed the effectiveness of these new doses for treating overweight patients which caused more improvement in their conditions with the rates of bleeding remaining the same [9-10].

In comparison to the scope of weight, other studies have shown that underweight patients with normal weight doses were considered to be high doses [7]. Furthermore, in a study that was published in 2016 by Carter et al., they discussed that there was not enough research on underweight patients that was targeted to legitimize the influence of higher anticoagulated risk. However, the result implied that underweight patients perhaps have been given the guideline doses, on the other hand, lower doses could be harmful rather than being beneficial to their condition [7]. Due to the lack of studies that would modify the anticoagulant guidelines to include weight variation to have efficient outcomes, our results have shown that only six patients (15%) who had adjusted VTE prophylaxis dose which is 2500 U Q12. Conversely, the majority of our sample size is given the standard dose regime of 5000 U Q12 and 5000 U Q8. The current practice and prescribing doses to the underweight patients is not highlighted enough for the healthcare provider to implement a standard dose to the target group; on the contrary, due to the unclear recommendation on how to identify the risk or the management causes these variations of the prescribing approach [11].

In recent literature, they propose that reduction of the VTE prophylaxis dose is active and safe in critically ill underweight patients, and it can constrict the risk of bleeding in those patients [12-15]. The previous context is supported by our study for the reason that among the patients who were given 2500 U Q12 which is the adjusted dose, our study has recorded zero complications of VTE and bleeding. Yet, 21 critically ill patients (52.50%) that were recorded in our study received the standard dose of 5000 U Q12 and one of them did develop PE and three have suffered a drop in hemoglobin which caused them to have a blood transfusion with no major bleeding episodes. Additionally, 13 patients were listed to be given 5000 U Q8 which caused one of these critically ill underweight patients to have DVT as a complication and another patient had a drop in hemoglobin which needed a blood transfusion.

Despite the findings reported in this study, there were a number of limitations that occurred in our study that was recorded: The low number of our sample size was due to the difficulties of our hospital system to extract patients based on their weight and their admission. Another limitation is that the number of patients who were considered underweight in the ICU is very limited which may be the reason that showed up as a result of insignificance in our analysis. In addition, the use of substances other than heparin as VTE prophylaxis also played a role in reducing our sample. Moreover, there was a frequent change of the doses due to the consistent change of the patient’s location as a result of the change in the patient’s health status; it resulted in moving the patient into different departments in the hospital, which resulted in not recording constant doses to be tracking the risks. It all contributed to the low sample size. 

## Conclusions

Venous thromboembolism is known recently to be one of the most common causes of death in critically ill admitted patients. Furthermore, to prevent this event from occurring, standard dosing of anticoagulants was used throughout the years; however, no adjustment of dosing has been made for some special circumstances. In this study, we described the pattern of heparin doses as VTE prophylaxis in underweight patients. Also, the standard dosing and adjusted dosage of VTE prophylaxis on underweight patients and any complications were compared. There was no significant difference in the complication’s outcome or benefits between the two groups.
